# Sociodemographic and Structural Risk Factors for Dengue in a Rapidly Developing Indonesian District

**DOI:** 10.3390/ijerph23060796

**Published:** 2026-06-14

**Authors:** Inke Nadia Diniyanti Lubis, Nelli Khalilah Sari Siregar, Gema Nazri Yanni, Isti Ilmiati Fujiati, Lenni Evalina Sihotang

**Affiliations:** 1 Faculty of Medicine, Universitas Sumatera Utara, Medan 20155, Indonesia; nellikhalilah23@gmail.com (N.K.S.S.); gema.nazri.yanni@usu.ac.id (G.N.Y.); isti@usu.ac.id (I.I.F.); lenni_sihotang@yahoo.co.id (L.E.S.); 2 Prof. Dr. Chairuddin P. Lubis Universitas Sumatera Utara Hospital, Medan 20154, Indonesia; 3 Department of Internal Medicine, Adam Malik Hospital, Medan 20137, Indonesia

**Keywords:** dengue, risk factors, semi-urban, environment, Indonesia

## Abstract

**Highlights:**

**Public health relevance—How does this work relate to a public health issue?**

- Dengue transmission is expanding beyond traditional urban centres into semi-urban and rural settings undergoing rapid urbanisation, yet determinants in these areas remain poorly understood.
- This study identifies household-level sociodemographic and environmental risk factors that directly contribute to dengue transmission in a rapidly developing Indonesian district.

**Public health significance—Why is this work of significance to public health?**

- This study demonstrates that dengue risk in transitional settings is driven by modifiable structural factors alongside demographic exposure patterns.
- This study also highlights a shifting epidemiology, with increasing transmission in semi-urban and rural populations, signalling gaps in current urban-focused prevention strategies.

**Public health implications—What are the key implications or messages for practitioners, policy makers and/or researchers in public health?**

- The findings support integration of low-cost housing improvements, environmental management, and school-based interventions into dengue control programmes in rapidly urbanising districts.
- Emphasises the need for multisectoral policies linking urban planning, vector control, and public health surveillance to address dengue transmission dynamics.

**Abstract:**

Background: Dengue infection is an expanding public health threat in Indonesia, increasingly reported in semi-urban areas undergoing rapid demographic and environmental change, where household-level determinants remain poorly characterised. Methods: We conducted a case–control study in the Deli Serdang district, North Sumatra, evaluating sociodemographic and environmental risk factors for dengue. Patients admitted to the district referral hospital (July–September 2024) were screened via medical records. Laboratory-confirmed dengue cases were compared with non-dengue febrile controls. Housing conditions and sociodemographic characteristics were assessed using a validated electronic questionnaire with photographic documentation. Multivariable logistic regression identified independent risk factors. Results: Of 238 individuals screened, 39 dengue cases and 78 controls were enrolled. Male sex (aOR 6.7, 95% CI 1.3–33.7), student status (aOR 7.8, 95% CI 1.1–56.5), absence of window screens (aOR 12.9, 95% CI 3.1–53.8), and surrounding vegetation (aOR 7.3, 95% CI 1.7–31.9) were independently associated with dengue infection. Rural residence was overrepresented among cases, suggesting expansion beyond traditional urban boundaries. Conclusions: Dengue risk in a transitional setting is shaped by demographic exposure and modifiable structural vulnerabilities. Integrated prevention strategies, including window screening, covered water storage, environmental management, and school-based vector control, are needed in rapidly urbanising districts.

## 1. Introduction

Dengue is a rapidly expanding mosquito-borne viral infection caused by four antigenically distinct serotypes of dengue virus and transmitted primarily by *Aedes aegypti* [1]. Globally, approximately half of the world’s population is at risk, with an estimated 100 to 400 million infections occurring annually. The global burden has increased dramatically over the past three decades, with incidence doubling approximately every decade since 1990. Recognising its escalating impact, dengue is included among the 20 priority conditions in the WHO 2021–2030 Neglected Tropical Diseases roadmap [2,3].

At the regional level, Southeast Asia bears a disproportionate share of the global dengue burden, with the highest transmission intensity concentrated in tropical and subtropical nations including Indonesia, the Philippines, Vietnam, and Thailand. Modelling projections indicate that dengue incidence across Southeast Asia is expected to increase substantially under future climate and demographic scenarios, driven by rising temperatures, expanding vector habitats, and rapid urbanisation [3]. Indonesia reported 257,271 dengue cases in 2024—more than double the 114,720 cases recorded in 2023 [4]. Transmission has traditionally been associated with densely populated urban centres; however, the epidemiology in Indonesia is rapidly evolving. Expanding peri-urban settlements, infrastructure development, environmental transformation, and increasing population mobility are reshaping vector ecology and transmission dynamics [3,5,6,7]. Semi-urban districts, characterised by mixed land use, transitional housing structures, and heterogeneous socioeconomic conditions, are emerging as important yet understudied settings for dengue transmission. In the North Sumatra province, the Deli Serdang district is one of the province’s fastest-growing districts and has recorded consistently high dengue incidence rates (47.7–59.3 per 100,000 population from 2020 to 2022). Despite this shift, most epidemiological evidence from Southeast Asia continues to focus on major urban centres. Household-level risk factors in semi-urban and transitioning districts remain poorly characterised, limiting the development of context-specific interventions.

We therefore conducted a case–control study to evaluate sociodemographic and environmental risk factors associated with dengue infection in the Deli Serdang district, a rapidly urbanising semi-urban district in North Sumatra, Indonesia. By focusing on a transitional ecological setting, this study aims to generate evidence to inform targeted, structurally grounded dengue prevention strategies in similar emerging districts across Southeast Asia.

## 2. Materials and Methods

**Figure 1:**
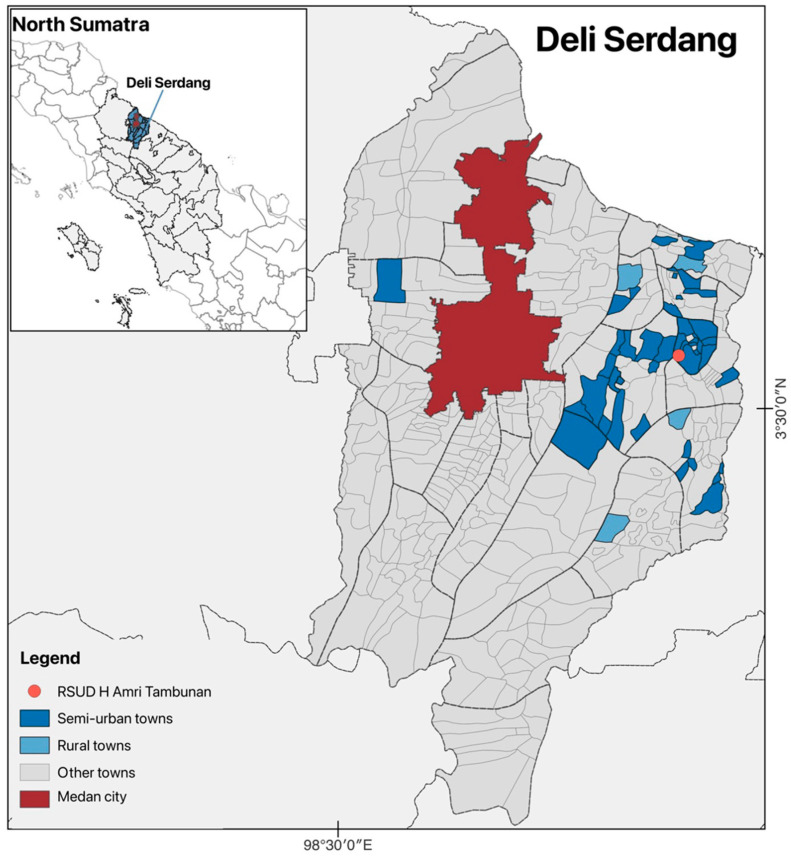
Map of the Deli Serdang district, North Sumatra, Indonesia.

This case–control study was conducted in the Deli Serdang district, located in North Sumatra Province, Indonesia (Figure 1). The Deli Serdang district is one of 33 districts in the province with an increasing trend of dengue cases. The district consists of 22 subdistricts and has a population of approximately 2.04 million. It has a tropical climate characterised by high rainfall, creating favourable conditions for the breeding of *Aedes aegypti* mosquitoes—the primary vector of the dengue virus. As a result, there were 974, 803, and 1210 reported cases in 2020, 2021, and 2022, respectively [8,9,10]. Based on the district population of approximately 2.04 million, the calculated dengue incidence rates were 47.7, 39.4, and 59.3 per 100,000 population in 2020, 2021, and 2022, respectively, indicating a persistent and substantial dengue burden in the district.

The participants were identified through medical records at the Drs. H. Amri Tambunan District Hospital, a referral hospital for Deli Serdang district. The study period (July to September 2024) was selected based on the availability of the hospital’s patient database and aligned with a period of elevated dengue activity at the district hospital, coinciding with Indonesia’s national dengue surge in 2024. Patients admitted between July and September 2024 who met the inclusion criteria were invited to participate in the study. Inclusion criteria for cases were: (1) a laboratory-confirmed diagnosis of dengue infection (positive NS1 antigen, IgM, or IgG antibody test); (2) residence within the Deli Serdang district; and (3) aged one year or older. Exclusion criteria (both groups) included: (1) more than one eligible individual identified from the same household; and (2) failure to provide informed consent. For controls, the inclusion criteria were hospital admission during the same period with febrile illness but without a diagnosis of dengue infection and residence in the Deli Serdang district. Both cases and controls were selected using purposive sampling until the target sample sizes of 39 and 78 were achieved. Participants were withdrawn if they could not be reached via WhatsApp within three contact attempts after enrolment, or if they completed less than 90% of the questionnaire items.

Demographic data were collected from the medical records. The enrolled subjects were contacted via WhatsApp to request their consent to participate in the study. Those who agreed to participate completed a validated questionnaire administered using an online electronic form. Parents or guardians of children aged less than 18 years were asked for their consent to complete the questionnaire. The questionnaires included information on housing conditions and environmental factors, with supporting photographic documentation of housing conditions to complement the survey data. Subdistricts were classified into urban, semi-urban, and rural areas based on population density, proportion of agricultural households, and access to city facilities according to the Central Statistics Bureau [11]. The questionnaire (Supplementary File S1) was developed based on a social-ecological framework for dengue transmission, encompassing four domains: (1) demographic and socioeconomic characteristics; (2) housing structure; (3) environmental conditions; and (4) household dengue history. Content validity was assessed by a panel of three experts in public health, infectious disease, and epidemiology. The validity was further confirmed through pilot testing with 10 participants not included in the main study. The home environmental conditions assessed included: distance between houses (<40 m vs. ≥40 m); roof structure (presence or absence of a ceiling beneath the roof); house wall type; floor material; household density (classified as high if more than four individuals resided in a household with floor area ≤ 36 m^2^ [12]); water storage container type (open vs. closed); window screen availability; presence of decorative plants; and presence of surrounding vegetation (trees, shrubs, or dense plant cover within 10 m of the dwelling perimeter). Photographic documentation was used to verify reported housing conditions.

All statistical analyses were performed using Stata software (version 17.0; StataCorp, College Station, TX, USA) [12]. Univariate analysis was used to describe the frequency and percentage distribution of the study variables, as well as the sociodemographic characteristics and environmental conditions. A bivariate analysis was performed to test the relationship between all independent and dependent variables (dengue incidence). The chi-square test was used with a significance level of 0.05. The association was expressed as an odds ratio (OR) with a 95% confidence interval (CI). Variables with a *p*-value ≤ 0.05 were considered to have a statistically significant relationship. Multivariate logistic regression analysis was performed to determine the influence of the most dominant variables on dengue incidence.

## 3. Results

In total, 238 participants were screened during the 3-month period, comprising 139 patients with dengue and 99 patients without dengue. Among the dengue cases, 47 patients (33.8%) resided outside the Deli Serdang district, 11 (7.9%) were untraceable, and four (2.9%) declined to provide consent; these individuals were excluded from the case group. After 39 eligible participants were enrolled, further recruitment to the case group was discontinued. In the non-dengue febrile illness group, 7 (7%) declined to participate and 14 (14.1%) could not be contacted, resulting in the enrolment of 78 participants as controls. Participants were enrolled from 5 villages and 38 towns spread across 10 subdistricts, with the majority of both dengue cases and non-dengue febrile controls residing in two subdistricts: Lubuk Pakam and Beringin. Table 1 shows the demographic characteristics of the two groups. The median ages of the dengue and the non-dengue febrile illness groups were 19 years (IQR 12–45 years) and 23.5 years (IQR 6–45 years), respectively. A significantly higher proportion of males (OR 3.017, 95% CI 1.357–6.710, *p* = 0.006) was observed among dengue cases than among non-dengue febrile controls. In addition, a greater proportion of students was identified among dengue cases (38.5%, OR 6.34, 95% CI 2.31–17.4, *p* ≤ 0.01). Surprisingly, rural residence was associated with higher odds of dengue compared with semi-urban residence (OR 0.08, 95% CI 0.009–0.78, *p* = 0.03), although the estimate was based on very small rural numbers. History of a household member with dengue infection was also associated with increased risk of acquiring dengue (OR 4.2, 95% CI 1.8–9.7, *p* = 0.001) than other causes of febrile illnesses. A larger proportion of dengue cases had lower educational levels (59% vs. 44.9%); however, the difference was not statistically significant (*p* > 0.05).

Table 2 presents the housing characteristics of the dengue cases and non-dengue febrile illness controls. The majority of participants live in a permanent house (98%), with ceilings (87%) and cement floorings (72%), having an open water container (58%), and house distance less than 40 m (79%). These housing conditions such as a distance between houses of less than 40 m (OR 4.4, 95% CI 1.2–15.9, *p* = 0.029), the absence of a ceiling beneath the roof (OR 7.3, 95% CI 2.1–24.7, *p* = 0.001), the presence of an open water container (OR 3.5, 95% CI 1.5–8.4, *p* = 0.007), and the absence of window screens (OR 8.9, 95% CI 3.7–21.6, *p* = 0.001), were significantly associated with increased risk of dengue infection than other febrile illnesses. In addition, the presence of decorative plants (OR 2.7, 95% CI 1.2–6.1, *p* = 0.022) and vegetation surrounding the house (OR 5.3, 95% CI 2.3–12.5, *p* = 0.001) were also associated with a higher odds of having dengue infection. In contrast, housing characteristics such as wall and floor types, and household density, did not significantly increase the risk of having dengue infection. In the multivariate analysis, only male gender (aOR 6.7, 95% CI 1.3–33.7, *p* = 0.021), being a student (aOR 7.8, 95% CI 1.1–56.5, *p* = 0.042), absence of window screens (aOR 12.9, 95% CI 3.1–53.8, *p* < 0.001), and presence of vegetation around the house (aOR 7.3, 95% CI 1.7–31.9, *p* = 0.008).

## 4. Discussion

Deli Serdang district represents a rapidly transforming district in North Sumatra Province, where urban expansion, population growth, and environmental change are occurring simultaneously. Our findings demonstrate that dengue transmission in this semi-urban setting is shaped by a combination of demographic vulnerability and modifiable structural and environmental factors.

We observed that male sex and student status were independently associated with dengue infection compared with non-dengue febrile illness. These findings likely reflect behavioural and mobility-related exposure patterns. In semi-urban districts, adolescent and young adults are frequently involved in frequent outdoor activities and occupational exposures, especially during the day, increasing daytime exposure to Aedes mosquitoes. The elevated risk among students aligns with regional studies from other Southeast Asian countries demonstrating increased dengue susceptibility in individuals aged 15–25 years, particularly in areas experiencing epidemiological transition [13,14,15,16,17]. Consistent with our findings, a case–control study in Hanoi, Vietnam, identified male sex and younger age as independent risk factors for dengue haemorrhagic fever [14], while a community-based study in Ho Chi Minh City similarly reported higher dengue risk among working-age males with frequent outdoor exposure [15]. In Thailand, Nakkhara et al. found that symptomatic dengue was associated with outdoor occupational and recreational activities, particularly among school-age individuals [18]. These converging findings across diverse Southeast Asian settings suggest that behavioural exposure patterns—rather than biological susceptibility alone—are a primary driver of the male and student predominance observed in dengue case series.

Importantly, this pattern may reflect an evolving immunity landscape. As dengue expands into newly urbanising and previously rural subdistricts, younger populations may lack prior exposure and protective immunity, rendering them more susceptible during outbreaks [17]. This observation underscores the need for school-based vector control strategies and targeted risk communication campaigns in semi-urban districts.

One of the most notable findings was the disproportionate representation of rural residents among dengue cases compared with non-dengue febrile controls. Although based on small numbers, this finding suggests that dengue transmission is no longer confined to urban centres but is encroaching into rural and peri-urban environments. This observation mirrors broader trends across Southeast Asia, where infrastructure development, road connectivity, agricultural transformation, and human mobility facilitate viral dissemination into previously low-transmission settings [19,20]. A systematic review by Kolimenakis et al. [20] demonstrated that urbanisation drives *Aedes* mosquito spread through the creation of artificial breeding sites, modified microclimates, and increased human density—processes now increasingly documented in peri-urban and transitional settings beyond traditional city centres. Dhewantara et al. [21] similarly reported that dengue in Indonesia has undergone significant spatial expansion between 2010 and 2017, with previously low-burden rural districts recording increasing incidence over time, consistent with our observation of rural case overrepresentation. In Indonesia, rapid land-use change and mixed settlement patterns blur the distinction between urban and rural ecologies, creating ideal breeding conditions for Aedes mosquitoes.

Among all environmental variables, the absence of window screens emerged as the strongest independent structural risk factor. The lack of window screens increases the likelihood of mosquitoes entering households, and unlike broader ecological drivers, window screening represents a modifiable and scalable intervention. Semi-urban households often have partial structural development, cement walls and permanent roofing but incomplete protective features such as screening or sealed ceilings. These transitional housing characteristics may inadvertently facilitate mosquito entry and indoor resting. Our findings suggest that relatively low-cost structural modifications could substantially reduce dengue risk. This is supported by experimental evidence: cluster-randomised controlled trials conducted in Merida and Acapulco, Mexico, demonstrated that the installation of insecticide-treated screens on doors and windows produced significant and sustained reductions in indoor *Aedes aegypti* density over a two-year follow-up period [22]. A systematic review and meta-analysis of housing interventions for Aedes-transmitted diseases similarly concluded that structural modifications, including screened openings, are among the most effective household-level measures for reducing vector entry [23]. These findings underscore the potential for window screening as a priority intervention in semi-urban settings where households are structurally incomplete.

Vegetation surrounding homes was also independently associated with infection. Vegetation modifies microclimate by increasing humidity and shading, conditions favourable for mosquito survival, particularly *Aedes albopictus*, which is more commonly associated with peri-urban and rural ecologies. The coexistence of *Aedes aegypti* and *Aedes albopictus* in transitional districts may alter vector dynamics and transmission intensity, a phenomenon increasingly observed in Southeast Asia [24,25]. It is important to note, however, that not all vegetation poses equal risk. Certain cultivated plants, particularly lemongrass (*Cymbopogon nardus*) and other aromatic herbs, contain essential oils (e.g., citronellal, geraniol) that have demonstrated mosquito-repellent properties. Citronella-based formulations have been shown to reduce *Aedes aegypti* landing rates by approximately 40% in laboratory and field conditions [26]. The risk associated with vegetation in our study therefore likely reflects unmanaged natural vegetation—trees, shrubs, and undergrowth providing humid, shaded resting habitats—rather than deliberately cultivated repellent plants. Promoting the selective cultivation of mosquito-repellent plants in household gardens may represent a complementary, low-cost vector control strategy worthy of further evaluation. These findings collectively highlight the need to integrate vector ecology into urban planning and environmental management strategies in rapidly developing districts.

Deli Serdang is one of the fastest growing districts in North Sumatra, with its population increasing from 1,790,431 in 2010 to an estimated 2,078,046 in 2025 [27]. The number of semi-urban areas almost doubled from 125 towns (31.6%) in 2010 to 233 towns (58.8%) in 2020 [11]. Despite this urban expansion, around 60% of adult participants in both dengue case and non-dengue febrile control groups belonged to low-socioeconomic households, with a monthly income below the district’s monthly minimum wage of USD 213 (IDR 3,500,000). Although income and education were not independently significant in multivariable analysis, these findings have important implications for dengue prevention. The high proportion of low-income households in both groups underscores the structural vulnerability of this population. Economic constraints limit access to preventive measures such as window screening, secure water storage, and environmental management. Previous studies have demonstrated strong associations between low socioeconomic status and dengue incidence, underscoring the importance of understanding how human behaviour and financial capacity influence transmission risk [28,29]. Our findings support several specific, actionable recommendations. First, Indonesia’s national 3M Plus programme (Menutup, Menguras, Mendaur ulang)—which promotes covering water containers, draining open water bodies, and recycling unused containers—should be reinforced and expanded across semi-urban and peri-urban settings in Deli Serdang, with particular attention to households lacking window screens and using open water containers. Second, local governments should consider subsidising window screening as a low-cost structural intervention for low-income households; evidence from cluster-randomised controlled trials in Mexico demonstrated sustained reductions in indoor *Aedes aegypti* density following insecticide-treated screen installation [30]. Third, school-based vector control programmes—including dengue prevention curriculum integration and student-led household inspections—should be expanded, given the disproportionate risk observed among students in this study. Finally, urban planning regulations in rapidly developing districts should incorporate mosquito habitat reduction as a standard requirement for new residential development, including mandatory vegetation management and covered water storage infrastructure.

This study had several limitations. First, sample selection was based on the patient database of the Drs. H. Amri Tambunan District Hospital, which may have introduced selection bias. Although the hospital serves as a referral centre for the entire Deli Serdang district, some subdistricts are geographically distant from this referral hospital. Access to care is also available at private hospitals and clinics under Indonesia’s universal health coverage scheme, which may capture these populations, as well as those with asymptomatic to mild infections. While we enrolled participants from nine of the 22 subdistricts, we could only confirm the presence of dengue in both semi-urban and rural areas without being able to accurately estimate the disease burden in each locality. Second, the large geographical area of Deli Serdang (2498 km^2^) limits our ability to conduct direct household and environmental assessments. Data collection via WhatsApp and electronic forms limited the depth and consistency of responses, although it allowed broader geographic coverage. Future studies should consider mixed-mode data collection to minimise non-response bias. Third, this study did not include entomological surveys of mosquito population density, which would have enabled direct correlation between vector abundance and human infection risk. Future studies in similar transitional settings should integrate entomological surveillance with human epidemiological data to better characterise the vector-human interface. Finally, the study was conducted over a three-month period (July to September 2024), which may not have captured a transmission peak. However, Sumatra follows an equatorial rainfall pattern with two wet season peaks annually, suggesting that dry season suppression of dengue transmission may be less pronounced in North Sumatra than in Java. Active dengue cases during the July September period were therefore plausible, particularly given Indonesia’s national surge in 2024 [31]. Nonetheless, a longer surveillance period is necessary to better understand the temporal dynamics of dengue transmission in the district. Future research should investigate additional environmental and behavioural determinants in transitional settings, including: longitudinal entomological monitoring using standardised larval and adult vector indices; GPS-linked geospatial mapping of case clustering; assessment of knowledge, attitudes, and practices regarding dengue prevention; and evaluation of water and sanitation infrastructure as structural determinants of mosquito breeding in rapidly developing communities. Moreover, dengue cases declined in 2025 (161,752 cases nationally) compared to the 2024 peak, though they remained above pre-surge 2023 levels, suggesting that the epidemiological transition in Indonesia is ongoing rather than resolved [32]. As this study was conducted over a short window in a dynamically changing district, continuous surveillance integrating updated urban expansion data would be necessary to track longitudinal trends in dengue epidemiology.

## 5. Conclusions

Dengue transmission involves complex interactions among the host, the virus, the vector, and the environment. Although this study did not capture all possible variables, it provides important insights into the sociodemographic and environmental determinants of dengue infection in rapidly urbanising districts. As semi-urban expansion occurs without parallel improvements in infrastructure and sanitation, dengue risk may disproportionately affect socioeconomically disadvantaged households. Addressing dengue in transitional districts therefore requires multisectoral approaches—combining housing improvement, infrastructure planning, and behavioural interventions.

## Figures and Tables

**Table 1:** Baseline characteristics.

Characteristics	Case *N* = 39 (%)	Control *N* = 78 (%)
Age, years		
Median (IQR)	19 (12–45)	23.5 (6–45)
<18	18 (46.2)	36 (46.2)
18–30	7 (17.9)	11 (14.1)
31–45	5 (12.8)	13 (16.7)
46–60	8 (20.5)	7 (9.0)
>60	1 (2.6)	11 (14.1)
Gender		
Male	25 (64.1)	29 (37.2)
Education		
Lower education *	23 (59.0)	35 (44.9)
Occupation		
Unemployed	11 (28.2)	38 (48.7)
Students	15 (38.5)	7 (9.0)
Farmers/labourers	4 (10.3)	11 (14.1)
Others	9 (23.1)	22 (28.2)
Income		
Below monthly minimum wage **	31 (79.5)	59 (75.6)
History of household member infected with dengue		
Yes	29 (74.4)	32 (41.3)
Subdistrict		
Lubuk Pakam	17 (43.6)	43 (55.1)
Tanjung Morawa	8 (20.5)	10 (12.8)
Pantai Labu	1 (2.6)	3 (3.8)
Beringin	5 (12.8)	11 (14.1)
Galang	5 (12.8)	6 (7.7)
Pagar Merbau	0 (0)	2 (2.6)
Batang Kuis	3 (7.7)	1 (1.3)
STM Hilir	0 (0)	1 (1.3)
Sunggal	0 (0)	1 (1.3)
Type of residential areas ***		
Semi-urban	34 (87.2)	53 (98.7)
Rural	5 (12.8)	1 (1.3)

* Lower education: elementary and middle school. ** District monthly minimum wage: IDR 3,500,000 (USD 213). *** Types of residential areas were classified according to data from the Central Bureau of Statistics (Indonesia) [11].

**Table 2:** Characteristics of home environmental conditions.

Characteristics	Case *N* = 39 (%)	Control *N* = 78 (%)	OR (95% CI)	*p*
Distance between houses			4.4 (1.2–15.9)	0.029
<40 m	36 (92.3)	57 (73.1)		
>40 m	3 (7.7)	21 (26.9)		
Roof			7.3 (2.1–24.7)	0.001
No ceiling	11 (28.2)	4 (5.1)		
With ceiling	28 (71.8)	74 (94.9)		
House wall			2.0 (0.1–33.3)	1.000
Semi-permanent	1 (2.6)	1 (1.3)		
Concrete	38 (97.4)	77 (98.7)		
Floor material			1.7 (0.8–4.0)	0.276
Cement	14 (35.9)	19 (24.4)		
Ground	25 (64.1)	59 (75.6)		
Household density *			1.7 (0.4–6.6)	0.713
High	4 (10.3)	5 (6.4)		
Low	35 (89.7)	73 (93.6)		
Water storage container			3.5 (1.5–8.6)	0.007
Open	30 (76.9)	38 (48.7)		
Closed	9 (23.1)	40 (51.3)		
Window screen			8.9 (3.7–21.6)	0.001
Not available	25 (64.1)	13 (16.7)		
Available	14 (35.9)	65 (83.3)		
Presence of decorative plants			2.7 (1.2–6.1)	0.022
Yes	26 (66.7)	33 (42.3)		
No	13 (33.3)	45 (57.7)		
Presence of vegetation around the house			5.3 (2.3–12.5)	0.001
Yes	21 (53.8)	14 (17.9)		
No	18 (46.2)	64 (82.1)		

* Household density was classified as high (dense) if more than four individuals resided in a household with a floor area of 36 m^2^ or less [11].

## Data Availability

The data presented in this study are available upon request from the corresponding author. The data are not publicly available due to privacy.

## Supplementary Materials

The following supporting information can be downloaded at: https://www.mdpi.com/article/10.3390/ijerph23060796/s1. File S1: Research Questionnaire.

## Abbreviations

The following abbreviations are used in this manuscript:

aOR	Adjusted Odds Ratio
OR	Odds Ratio
CI	Confidence Interval
IDR	Indonesian Rupiah
IgG	Immunoglobulin G
IgM	Immunoglobulin M
IQR	Interquartile range
m	metre
USD	United States Dollar
WHO	World Health Organization
