# Supplementary material for: Sociodemographic and Structural Risk Factors for Dengue in a Rapidly Developing Indonesian District
Source: Int J Environ Res Public Health. 2026 Jun 14;23(6):796. doi: 10.3390/ijerph23060796 (PMC13300014; doi:10.3390/ijerph23060796)
Supplement: Supplementary file 1 [file ijerph-23-00796-s001.zip › ijerph-4284880-supplementary.pdf]

RESEARCH QUESTIONNAIRE

Sociodemographic and Environmental Risk Factors for Dengue Fever in a Rapidly Developing Indonesian District

INSTRUCTIONS FOR PARTICIPANTS

This questionnaire is part of a research study on dengue fever risk factors in Deli Serdang District, North Sumatra. Your participation is voluntary and all responses are strictly confidential. Please answer all questions honestly and completely. If you have any questions, please ask the study interviewer.

For multiple-choice questions, **please tick (✓) one box only**, unless otherwise indicated.

SECTION A: PARTICIPANT INFORMATION

Age (years): ____

Gender: □ Male □ Female

Telephone Number: ____

Home Address: ____

Subdistrict (Kecamatan): ____

A1. What is your highest level of education completed?

- □ 1. College / Bachelor’s Degree or higher
- □ 2. Senior High School or equivalent (SMA/SMK)
- □ 3. Junior High School or equivalent (SMP)
- □ 4. Primary School or equivalent (SD)
- □ 5. No formal schooling

A2. What is your current occupation?

- □ 1. Civil servant / Government employee
- □ 2. State-owned enterprise (BUMN) employee
- □ 3. Self-employed / Entrepreneur
- □ 4. Labourer / Factory worker
- □ 5. Fisherman / Farmer
- □ 6. Homemaker / Housewife
- □ 7. Student
- □ 8. Unemployed
- □ 9. Other (please specify): ____________________________

A3. What is your approximate monthly household income?

- □ 1. Below the District Minimum Wage (UMK) — below Rp 3,500,000
- □ 2. At or above the District Minimum Wage (UMK) — Rp 3,500,000 or more
- □ 3. No income

SECTION B: HOME ENVIRONMENTAL CONDITIONS

B1. What is the approximate distance between your house and the nearest neighbouring house?

- □ Less than 40 metres
- □ 40 metres or more

B2. Does your house have a ceiling (plafon)?

- ◻ Yes
- ◻ No

B3. What is the current condition of the floor in your house?

- ◻ Unfinished / bare floor (wood, cement slab, or board without tiling)
- ◻ Finished / tiled floor (ceramic or similar)

B4. What type of construction best describes your house?

- ◻ Semi-permanent (e.g., raised/stilt house, wooden house, or bamboo house)
- ◻ Permanent (e.g., brick or concrete wall construction)

B5. What is the approximate floor area of your house (length × width)?

- ◻ 36 m^2 or less
- ◻ More than 36 m^2

B6. How many people currently live in your house?

- ◻ 4 or more people
- ◻ 1 to 3 people

B7. What type of water storage container do you use in your household?

Answer: ____

B8. What is the condition of the water storage container(s) you use?

- ◻ Open (uncovered)
- ◻ Closed (covered)

B9. Are mosquito wire screens (kawat nyamuk) installed on the ventilation openings, windows, or doors of your house?

- ◻ Yes, screens are installed
- ◻ No screens installed

If not installed, please state the reason:

Reason: ____

B10. Are there any ornamental plant pots (that could hold standing water) around or inside your house?

- ◻ Yes
- ◻ No

B11. Is there dense vegetation, shrubs, or trees surrounding your house (within 10 metres of the house perimeter)?

- ◻ Yes
- ◻ No

SECTION C: DENGUE FEVER HISTORY

C1. Have you ever been diagnosed with dengue fever by a doctor?

- ◻ Yes
- ◻ No

If yes, please answer questions C2 and C3.

C2. When were you most recently diagnosed with dengue fever?

- ◻ Less than 1 year ago
- ◻ More than 1 year ago

C3. How many times in total have you been diagnosed with dengue fever by a doctor?

- ◻ Once

- ☐ More than once

C4. Has any member of your immediate household ever been diagnosed with dengue fever by a doctor?

- ☐ Yes
- ☐ No

INTERVIEWER USE ONLY

Date of interview: ________________________________ Interviewer initials: ________________

Mode of administration: ☐ In-person ☐ Telephone ☐ Self-administered (online)

Notes:

____

Thank you for your participation.
